# Strength Evaluation of Functionalized MWCNT-Reinforced Polymer Nanocomposites Synthesized Using a 3D Mixing Approach

**DOI:** 10.3390/ma15207263

**Published:** 2022-10-18

**Authors:** Vijay Patel, Unnati Joshi, Anand Joshi, Ankit D. Oza, Chander Prakash, Emanoil Linul, Raul Duarte Salgueiral Gomes Campilho, Sandeep Kumar, Kuldeep Kumar Saxena

**Affiliations:** 1 Department of Mechanical Engineering, Parul University, Vadodara 391760, Gujarat, India; 2 Department of Mechatronics Engineering, Parul University, Vadodara 391760, Gujarat, India; 3 Department of Computer Sciences and Engineering, Institute of Advanced Research, Gandhinagar 382426, Gujarat, India; 4 School of Mechanical Engineering, Lovely Professional University, Phagwara 144411, Punjab, India; 5 Division of Research and Development, Lovely Professional University, Phagwara 144411, Punjab, India; 6 Department of Mechanics and Strength of Materials, Politehnica University Timisoara, 1 Mihai Viteazu Avenue, 300222 Timisoara, Romania; 7 Departamento de Engenharia Mecânica, Instituto Superior de Engenharia do Porto, Rua Dr. Bernardino de, 4249-015 Almeida, Portugal; 8 Division of Research Innovation, Uttaranchal University, Dehradun 248007, Uttarakhand, India; 9 Department of Mechanical Engineering, GLA University, Mathura 281406, Uttar Pradesh, India

**Keywords:** polymer nanocomposites, functionalization, multi-wall carbon nanotube, 3D mixing

## Abstract

The incorporation of carboxyl functionalized multi-walled carbon nanotube (MWCNT- COOH) into a polymethyl methacrylate (PMMA) has been investigated. The resultant tensile and flexural mechanical properties have been determined. In this paper, a novel synthesis process for a MWCNT-reinforced polymer nanocomposite is proposed. The proposed method significantly eliminates the most challenging issues of the nano-dispersed phase, including agglomeration and non-homogeneous mixing within a given matrix material, and also resolves the issues occurring in conventional mixing processes. The results of scanning electron microscopy support these claims. This 3D-mixing process is followed by an extrusion process, using a twin-screw extruder for pristine MWCNT, and a compression molding process for COOH-MWCNT, to prepare test specimens for experimentally determining the mechanical properties. The test specimens are fabricated using 0.1, 0.5, and 1.0 wt.% MWCNT, with a remaining PMMA phase. The testing is conducted according to ASTM D3039 and ASTM D7264 standards. Significant improvements of 25.41%, 35.85%, and 31.75% in tensile properties and 18.27%, 48%, and 33.33% in flexural properties for 0.1, 0.5, and 1.0 wt.% COOH-MWCNT in PMMA, respectively, compared to non-functionalized MWCNTs, were demonstrated. The highest strength was recorded for the nanocomposite with 0.5 wt.% f-MWCNT content, indicating the best doping effect at a lower concentration of f-MWCNT. The proposed CNT-PMMA nanocomposite may be found suitable for use as a scaffold material in the domain of bone tissue engineering research. This type of research possesses a high strength requirement, which may be fulfilled using MWCNT. Furthermore, this analysis also shows a significant amount of enhancement in flexural strength, which is clinically required for fabricating denture bases.

## 1. Introduction

The nanocomposite is a multiphase solid material where one of the phases possesses a nano level dimension. The final product does not need to be on the nanoscale, but can be macroscopic in size [1]. Based on the matrix material, nanocomposite materials are divided into three distinct groups: ceramic matrix nanocomposites (CMNC), metal matrix nanocomposites (MMNC), and polymer matrix nanocomposites (PMNC) [2]. Polymer is an adaptable material, exhibiting numerous extraordinary properties such as adaptability, low thickness, simple processibility, low cost, etc. In any case, the mechanical properties of these materials are lacking for some design and biomedical applications [3]. Because of the more valuable mechanical properties [4] regarding other known filler materials, the disclosure of carbon nanotubes (CNT) has attracted the extensive consideration of analysts and industries. CNT exists in two assortments because of various layers of graphene sheets, called single-walled carbon nanotubes (SWCNTs), with one ring and a variety of coaxial nanotubes, known as multiwall carbon nanotubes (MWCNTs) [5]. The biggest challenge in the manufacturing of nanocomposites containing CNTs is the lack of dispersion and orientations [6]. During the reinforcement of nanocomposites, nanotubes are easily agglomerated due to a strong intertube van der Waals attraction, hence limiting the effective use of their exceptional properties obtained at the individual level [7]. The combination of polymer nanocomposites typically applies bottom-up or top-down systems [8,9]. The preparation of polymer nanocomposites can be accomplished by various methods, including in-situ polymerization, solution blending, and melt intercalation. Poornima et al. [10] developed a nanocomposite with 1 wt.% carboxyl functionalized MWCNT in polypropylene (PP) using a Haake Extruder, followed by injection molding. Based on the investigation, they concluded, that the impact strength of prepared material increased with the addition of 1 wt.% functionalized MWCNT, whereas the tensile and flexural strength were reduced compared to pure PP. Adin [11] studied the effect of a reinforced particle on the compressive and bending properties of pipe prepared using structural epoxy adhesive and mica as the filler. The composite pipes were loaded axially for measuring bending and compressive strength, and the results show that the addition of filler enhanced both properties.

Kalaitzidou et al. [12] figured out how to obtain polypropylene nanocomposites built up with exfoliated graphite nanoplatelets by melt-blending polymer arrangement, followed by compression or injection molding. They performed the sonication within the presence of isopropyl alcohol (IPA) to further develop scattering and to acquire electrically conductive composites with a low permeation limit of 0.3 vol.%. Adin et al. [13] studied adhesives prepared using polyester and fiberglass powder as filler materials with 1 and 2 wt.% for use in the aviation field, and their mechanical properties were evaluated. Jindal et al. [14] experimented to evaluate the mechanical properties of MWCNT-polycarbonate nanocomposite prepared by solution blending, followed by compression molding. The correct selection of the preparation technique is critical in order to obtain nonmaterial with suitable properties [15,16,17]. The morphology and mechanical properties of the functionalized-MWNTs filled polymethyl methacrylate (PMMA) composite were studied via scanning electron microscopy and a universal testing machine [17,18]. Mehmet and Erol [19] presented the application of low-cost and lightweight adhesive joints in aerospace, aviation, and automotive applications compared to traditional jointing methods. To better resist the environmental effect, the mechanical and dimensional properties were enhanced with the use of glass fiber reinforcement in preparing the composite.

PMMA is non-toxic, and it exhibits good compressive resistance and good processing capabilities. PMMA is one of the most common polymers used in the manufacture of a wide range of dental applications, including the fabrication of artificial teeth, denture bases, dentures, orthodontic retainers, temporary or provisional crowns, and for the repair of dental prostheses. The improvement of the mechanical properties of PMMA continues to be one of the most actively researched domains in the field of dental biomaterials [20,21,22,23]. PMMA bone cement is an essential element of orthopedic component fabrication due to its magnificent biocompatibility and mechanical properties. It is extensively used in the biomedical sector as a synthetic bone material. The findings of mechanical testing and advanced characterization demonstrate the application potential for developing orthopedic implants in the biomedical sector. Various attempts have been made to modify and improve the mechanical properties by using different metal oxide fillers and fibers to include the broad scope of PMMA [24,25,26,27]. Vardharajula et al. [28] reported that the biocompatibility of a CNT-based composite mainly depends on the size, dose, duration, testing systems, and functionalization used. The surface modification improves their solubility and biocompatibility and alters their cellular interaction-reduced cytotoxic effects. Ma et al. [29] investigated the efficiency of CNTs as a reinforcement for polypropylene (PP) for biocompatible applications. The type of functional group may be the key factor affecting the mechanical properties and biocompatibility of PP nanocomposites compared to neat PP.

Hamit and Mehmet [30] investigated the tensile and bending strength of a composite produced in the form of epoxy adhesive layers by using a hand lay-up method in which aluminum, mica, and ceramic particles were added into epoxy as a structural adhesive by 2, 4 and 6 wt.% and achieved good enhancement in tensile strength and bending strength for 4 wt.% and 2 wt.%, respectively. Adin and Okumus [31] presented the microstructural characterizations of martensitic stainless steel and mild/low carbon steel welded by friction welding using an optical microscope (OM), X-ray diffractometer (XRD), and scanning electron microscope (SEM). Satish et al. [32] presented the synthesis of multi walled carbon nanotubes (MWCNT’s)/E glass fiber and their morphology, and the thermal behavior with different percent loadings of MWCNTs was inspected. The results showed that a clear improvement in the thermal stability of the composites increased with increasing MWCNTs content. Rouway et al. [33] performed homogeneous mixing using the Mori–Tanaka approach for CNT and GNP nanofillers, and then the matrix was used with alfa and E-glass isotropic fibers. They investigated the elastic properties with the influence of the volume fraction Vf and the aspect ratio AR of the nanofiller. The enhancement in properties was reported for CNT and GNP-reinforced nanocomposites, as well as a further increase in the volume fraction and aspect ratio of the nanofillers, Young’s modulus E increases and Poisson’s ratio ν decreases. Parnian and D’Amore [34] presented a novel process to prepare CNT-reinforced filaments using the sol gel method to improve the mechanical, thermal, and electrical properties with the homogeneous dispersion of CNTs in a dilute polymer solution. The optimizing the filament can also reasonably improve the orientation of CNT in the filament.

Based on the above-cited literature, it is quite evident that a PMMA-based nanocomposite has a vital role to play in various domains of the biomedical field. Moreover, the two main problems related to the synthesis of nanocomposites are related to the agglomeration of CNTs and the lack of interfacial adhesion. A 3D mixer is ideal for the homogeneous mixing of powder; however, this may not be the case with different bulk density, proportions of mixing, or shape and particle size. It is often observed that the particles with different densities or sizes form clusters on the outer periphery of the mixer or at the centre of the mixing mass. This is resolved by a rotary or shaking motion, which provides the required homogeneity. A 3D mixer exhibits an inversion motion rather than a rotary motion. Since there is no rotating blade inside, there is no shear force being applied to the material. The major problems of agglomeration can be solved by using a novel 3D mixing method. The characterization images presented in a later section indicate that the novel mixing process is free of agglomeration. In order to increase the interfacial adhesion, the authors have utilized functionalized MWCNT, achieving promising results, as detailed in the following sections.

## 2. Materials and Methods

The synthesis procedure, testing procedure, and characterization technique are presented in this section. The tensile strength and flexural strength are determined experimentally for different mixing ratios of functionalized CNTs with the PMMA matrix.

PMMA is a synthetic resin delivered from the polymerization of methyl methacrylate, commonly known as acrylic. It must be preheated before micro compounding in a vacuum oven at 60 °C for 1 h to remove its moisture content. PMMA, in its fine powder form, was obtained from N. Shashikant Co. Mumbai (Mumbai, India); its properties are shown in Table 1.

CNTs are tubes with nanometer measurements and micron lengths comprised of hollow cylinders of graphene with remarkable electronic and mechanical properties. This carbon nanotube has high carbon purity and length-to-diameter ratio. The maximum advantage of CNTs as the filler has been limited because of poor interfacial interaction and van der Waals connection among CNTs and the polymer lattice. To resolve such issues, the CNTs are made chemically more reactive by functionalizing them [35]. These approaches can be simply divided into chemical (covalent) and physical (noncovalent) functionalization as interactions between active materials and CNTs. Carboxyl-modified multi-walled carbon nanotubes were utilized for better dispersion of multi-walled carbon nanotubes into the polymer matrix. MWCNT-COOH was prepared by the oxidation of pristine (p) MWCNTs in a concentrated H_2_SO_4_/HNO_3_ mixture. Into a flask equipped with a condenser, p-MWCNTs, HNO_3_, and H_2_SO_4_ were added under vigorous stirring. Before the reaction, the flask was immersed in an ultrasonic bath for 10 min. Then the mixture was stirred for 100 min under reflux; the oil bath temperature was increased gradually from 90 °C to 133 °C. After cooling to room temperature, the reaction mixture was diluted with deionized water and then vacuum-filtered through filter paper. The dispersion, filtering, and washing steps were repeated until the pH of the filtrate reached 6–7. The filtered solid was dried under vacuum for 24 h at 60 °C, yielding MWCNT-COOH [36,37]. Carboxyl (COOH) is a functionalized group of carboxylic acid that strongly interacts with many organic and inorganic materials. MWCNTs were procured from Platonic Nanotech Pvt Ltd. (Jharkhand, India). The properties of MWCNTs are shown in Table 2.

## 3. Specimen Preparation Procedure

### 3.1. The 3D Mixing Process

Homogeneous dispersion of nanoparticles in a polymer by using traditional compounding techniques is very difficult due to the strong tendency of fine particles to agglomerate. A 3D mixer is ideal for the homogeneous mixing of powder; it may be more difficult with different bulk density, or proportions of mixing, or shape and particle size. The classical tumbling motion ensures that every particle of powder changes its position and mixes at every angle of motion, which reduce the clustering of materials with different densities on the outer periphery of the mixer, or at the center of the mixing mass [33]. The important parameters of a 3D mixture are rotor speed, mixing time, rotary direction, and filler loading. The interactions between these parameters are also vital. To evaluate this, the mixing parameters are changed simultaneously, and the data are analyzed using a visual inspection of the response of mixture. The results reveal that mixing time and rotor speed strongly affect the overall performance of the prepared composite. The experiment was performed for 0.1, 0.5, and 1 wt.% of MWCNT in PMMA polymer. The operating parameters for the 3D mixing process are speed, direction, and time, as shown in Table 3.

### 3.2. Extrusion and Injection Molding Process

**Figure 1:**
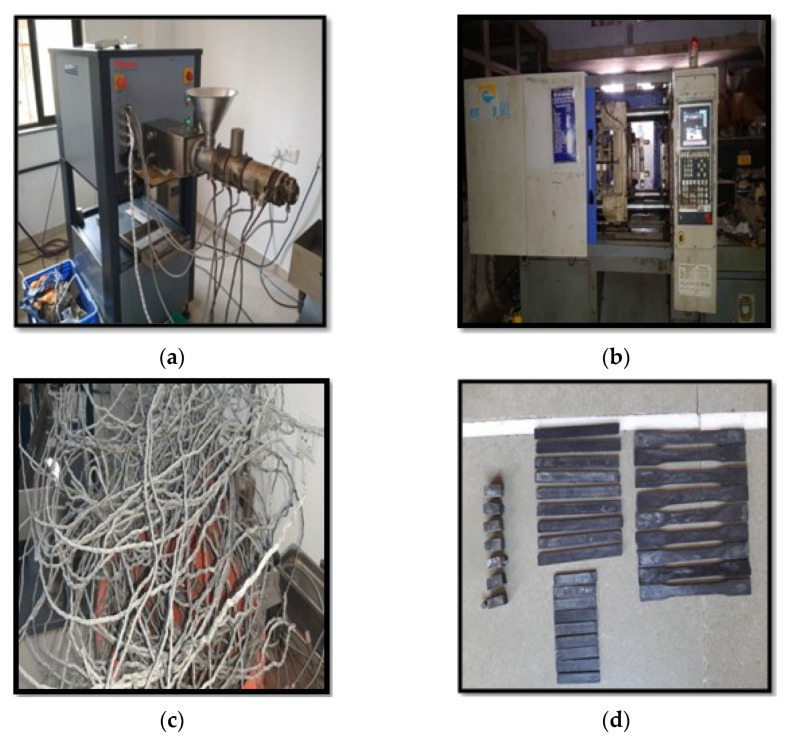
Specimen preparation of pristine MWCNT/PMMA: (**a**) twin screw extruder setup, (**b**) injection molding setup, (**c**) filament generation of the prepared nanocomposite, and (**d**) testing specimen preparation.

Before compounding, the mixer was preheated/dried at 80 °C in a vacuum oven for 6 h to remove moisture and to enable the uniform distribution of PMMA and CNTs particles, which will subsequently influence the nanocomposite homogeneity [6,35]. As shown in Figure 1, PMMA/MWNT nanocomposites were prepared with a co-rotating twin-screw extruder. The effect of the processing conditions and extruder characteristics on the dispensability of nanocomposites is investigated. Dried pellets of polymers were mixed in a container and melt-blended in an intermeshing, co-rotating, twin-screw extruder made by Thermo Scientific, followed by injection molding. The mixture is fed into the hopper, and it moves towards the barrel at a temperature of 240 °C, melting before reaching the bottom of the barrel. The mixture is fed into the barrel, which houses a twin co-rotating screw rotating at a speed of 50 rpm.

An injection molding machine is used for sample preparation. It is crucial to set proper parameters, particularly regarding thermal and shear loading. It is also necessary to avoid the degradation of the nanotube structure. The nozzle contains heating oil, which maintains the temperature of the compounded melt at around 230 °C. For production, an injection mold with a central ejector, which had exchangeable plates according to requirements, was used for testing samples. The holding pressure time was 35 s. The mold was cooled to a temperature of 50 °C for both halves of the injection mold. The compounded materials discharged from the micro compounder are accumulated inside the cylinder (nozzle). Pressure shaping is a notable assembling process for a composite. Specifically, the advancement of high-strength sheet molding compounds motivated the widespread adoption of the compression molding process in automotive and appliance applications [38,39,40]. In compression molding, the remeasured volume of the powder mixture is set directly into a heated mold cavity. The temperature of the mold is kept at more than 280 °C. The quantity of charge (molding material) put into the mold is calculated by considering the parameters mentioned in Table 4.

**Figure 2:**
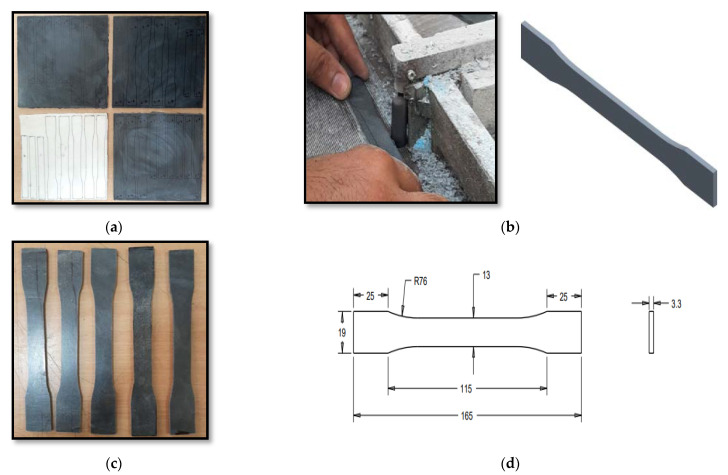
COOH-MWCNT/PMMA specimen preparation: (**a**) sheet generation using compression molding, (**b**) machining process of specimens, (**c**) obtained specimens, (**d**) specimen dimensions.

The samples were prepared by cutting/trimming them from compression-molded nanocomposite sheets, as per the ASTM D3039 [41] and ASTM D7264 [42] standards, as shown in Figure 2. The specimens were prepared as per ASTM standard regarding the size of specimen for a tensile test, having an overall dimension length of 165 mm, width of 19 mm, and thickness of 3.3 mm; the grip length, gauge length, and gauge width were 125 mm, 50 mm, and 13 mm, respectively.

### 3.3. Specimen Testing and Characterization

**Figure 3:**
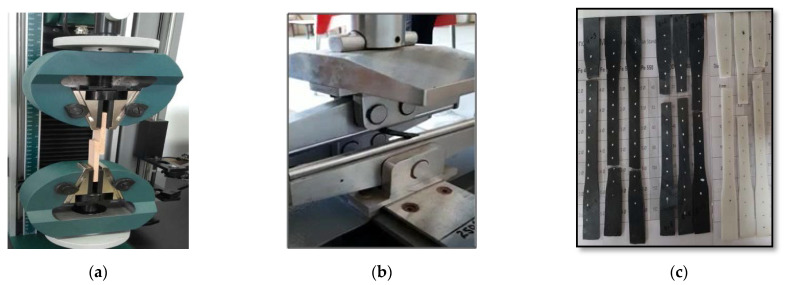
Specimen testing setup for (**a**) tensile test, (**b**) flexural test, and (**c**) tested specimens.

The properties of polymers are not only determined by their chemical structure, but also by the processing method, which determines the orientation of the filler in the final products [43]. The tensile properties of injection molded PMMA/MWCNT specimens were measured with a universal tension test machine at ambient conditions (room temperature and humidity) with a crosshead speed of 0.1 mm/min, according to the ASTM D3039 standard [41]. The flexural properties of nanocomposites were determined with the three-point bending test apparatus with a crosshead displacement rate of 1 mm/min, as per the ASTM D7264 standard [42]. The crucial parameters that determine the effects of fillers on the properties of composites are: filler size, shape, and aspect ratio, along with and filler-matrix interactions. The mechanical properties of the prepared specimens were evaluated with tensile and flexural tests, as shown in Figure 3. The dispersion and distribution of CNTs within the prepared nanocomposites can be observed using SEM [44,45,46,47]. The SEM images were obtained using SEM JSM-6010LA, Jeol LTD (Akishima, Tokyo, Japan).

## 4. Results and Discussion

### 4.1. Microstructural Assessment

**Figure 4:**
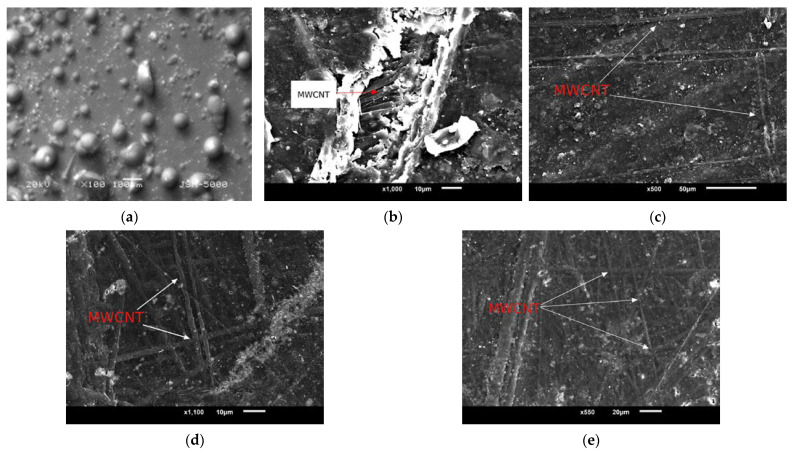
SEM images of (**a**) nanocomposite in powder form, (**b**) crack surface, (**c**) 0.1 wt.% COOH- MWCNT, (**d**) 0.5 wt.% COOH-MWCNT, and (**e**) 1.0 wt.% COOH-MWCNT in PMMA.

SEM images of the powder form of PMMA/MWCNT and fractured structure of 0.5 wt.% MWCNT filled PMMA nanocomposites are shown in Figure 4. In Figure 4c–e, the long and undamaged MWCNTs show that there is no breakage of MWCNTS during handling, which indicates that the 3D mixing process provided a positive impact on nanocomposite handling. It also provides the maximum advantage of the L/D ratio for property enhancement.

The morphological form of the tube is uniform, and the presence of clots and clumps around the MWCNT contain white spots. The white patches and dots are not representative of carbon nanotubes, but rather the polymer matrix used in the synthesis process. The size of the CNT’s diameter is estimated from an SEM image with 1000× magnification.

### 4.2. Mechanical Assessment

**Figure 5:**
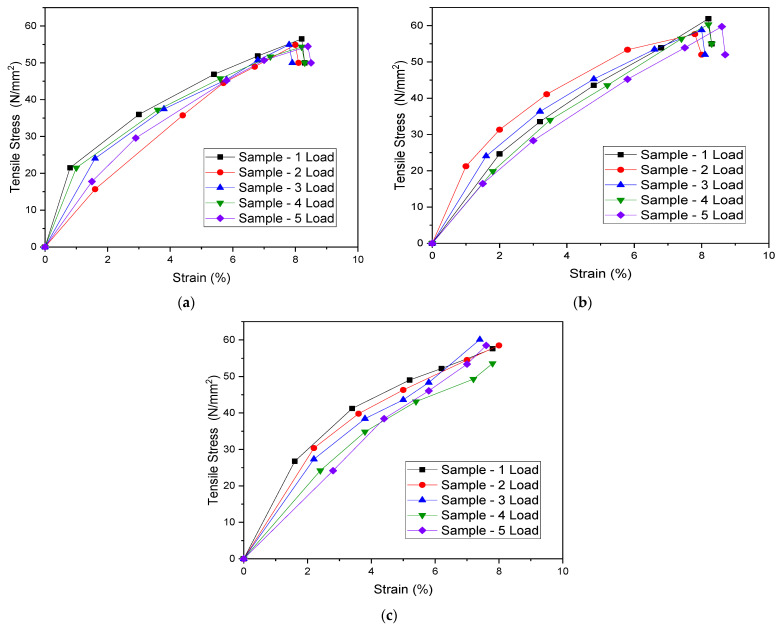
Tensile stress–strain curves for (**a**) 0.1, (**b**) 0.5, and (**c**) 1.0 wt.% COOH-MWCNT in PMMA.

As per ASTM standards for each combination, five specimens were tested, and their tensile and flexural test results are reported for 0.1, 0.5, and 1.0 wt.% MWCNT/PMMA, respectively. Figure 5 shows the stress–strain curves of the COOH-MWCNT/PMMA composite specimens, as a measurement of the evolution of the mechanical properties of the PMMA composites with f-MWCNT. The graph of tensile stress increased in a linear manner at the initial phase, showing the elastic deformation stage of the material. Later, the stress reaches its maximum value and starts decreasing up to the complete failure state. The presented graph clearly indicates that a combination of a linear and nonlinear regime is found when evaluating the tensile stress of the given specimen. Figure 5 exhibits a linear trend until it reaches a strain value of 1%, which tends to become nonlinear with the increase in the strain rate. The reason for this nonlinearity can be attributed to the behavioral aspect of the nanocomposite and the amount of reinforcement. A similar trend is also observed when the amount of reinforcement in the PMMA matrix is increased up to 1%.

**Figure 6:**
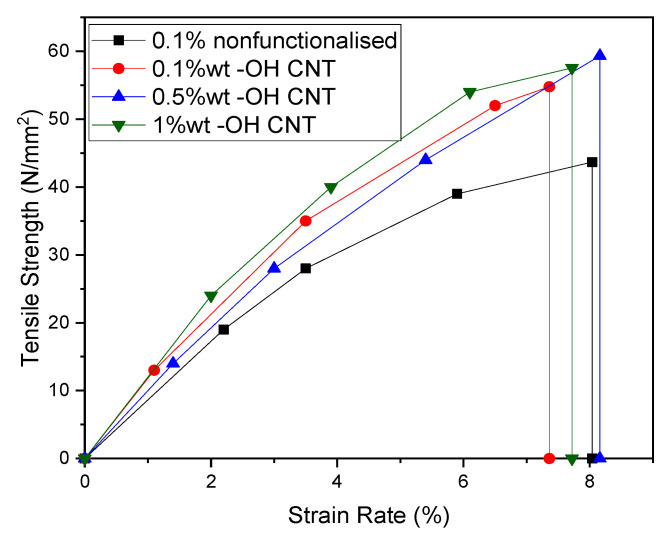
Tensile stress–strain curves for non-functionalized and f-MWCNT nanocomposites.

**Figure 7:**
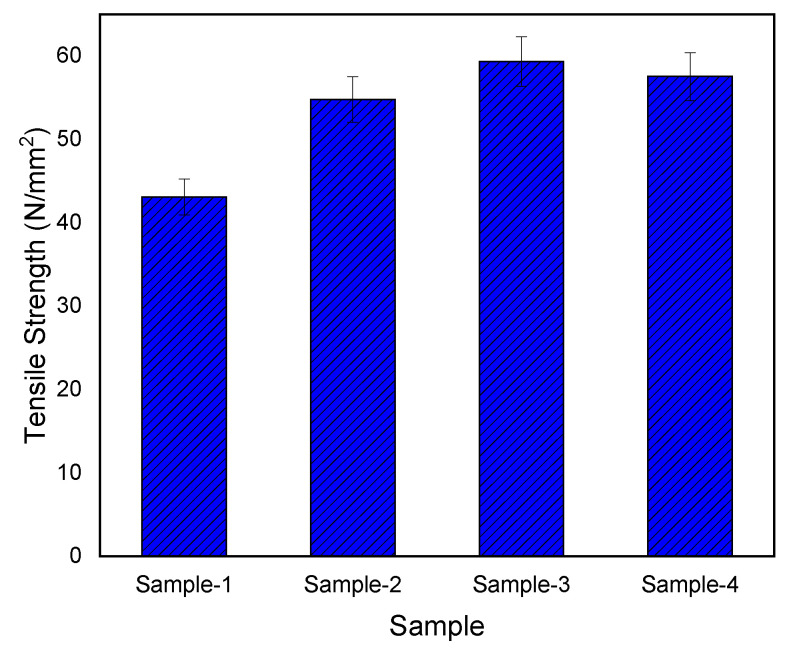
Tensile strength variation for non-functionalized (Sample-1: 0.1 wt.%) and functionalized (Sample-2: 0.1 wt.%; Sample-3: 0.5 wt.%; and Sample-4: 1.0 wt.%) MWCNT/PMMA nanocomposites.

The stress–strain curves for non-functionalized and f-MWCNT/PMMA nanocomposites are shown in Figure 6, and the impact of wt.% on tensile strength is highlighted in Figure 7.

The tensile strength significantly increases with the increasing amount of MWCNT up to 0.5 wt.% (Figure 6). The tensile strength of PMMA/MWCNT increased significantly as the MWCNT content was increased from 0.1 to 1.0 wt.%, from 43.68 MPa (pristine MWCNT) to 54.78 MPa (0.1 wt.%), 59.34 MPa (0.5 wt.%), and 57.55 MPa (1.0 wt.%), respectively. It is also noted that the addition of 0.5% f-MWCNT results in an enhancement in values of the maximum strength for PMMA to 59.34 MPa. Such improvement in the mechanical properties indicated excellent reinforcement of the f-MWCNTs in the PMMA matrix. That is, 3D mixing could play a synergetic role, not only in improving the dispersion of MWCNTs in the PMMA matrix, but also in strengthening the reinforcement due to the hyperactive branched polymer structure of the functionalized polyamide molecules [48]. The effect on tensile strength is 24.56%, 35.95%, and 31.75% for 0.1, 0.5, and 1.0 wt.% COOH-MWCNT in PMMA, respectively.

**Figure 8:**
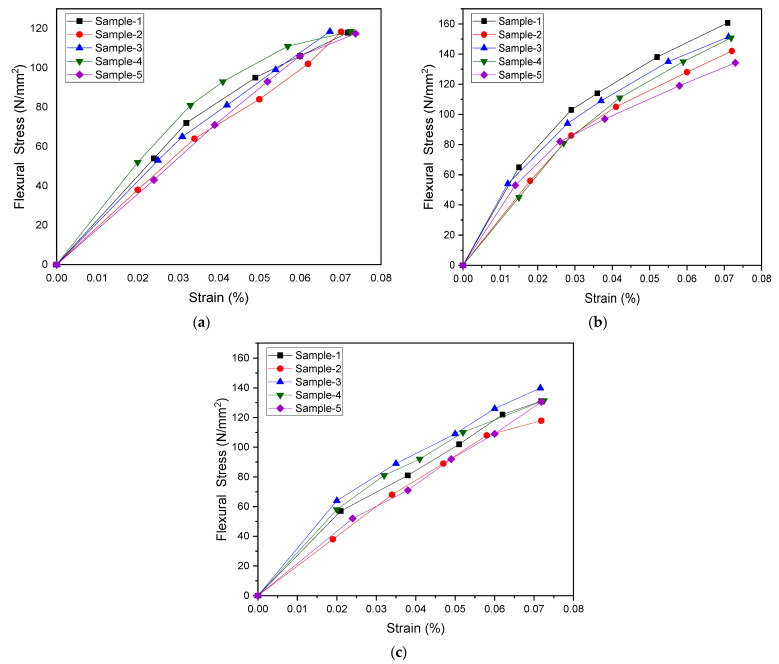
Flexural stress–strain curves for (**a**) 0.1, (**b**) 0.5, and (**c**) 1.0 wt.% COOH-MWCNT in PMMA.

Similarly for flexural testing, five samples of each mixing combination were tested, and the results are shown in Figure 8. Flexural strength significantly increases with the increasing amount of MWCNT up to 0.5 wt.%.

**Figure 9:**
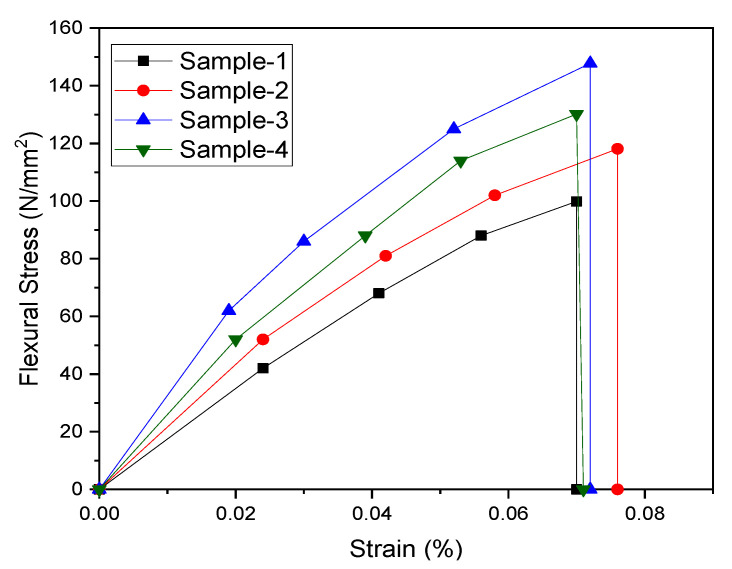
Flexural stress–strain curves for non-functionalized (Sample-1: 0.1 wt.%) and functionalized (Sample-2: 0.1 wt.%; Sample-3: 0.5 wt.%; and Sample-4: 1.0 wt.%) MWCNT nanocomposites.

The comparative stress–strain curves of non-functionalized and carboxyl functionalized MWCNT in PMMA polymer are shown in Figure 9. It is observed that the flexural stress increases for 0.1 and 0.5 wt.% and then decreases for 1.0 wt.%. This shows a sharp initial increase, followed by a slightly decreasing trend.

**Figure 10:**
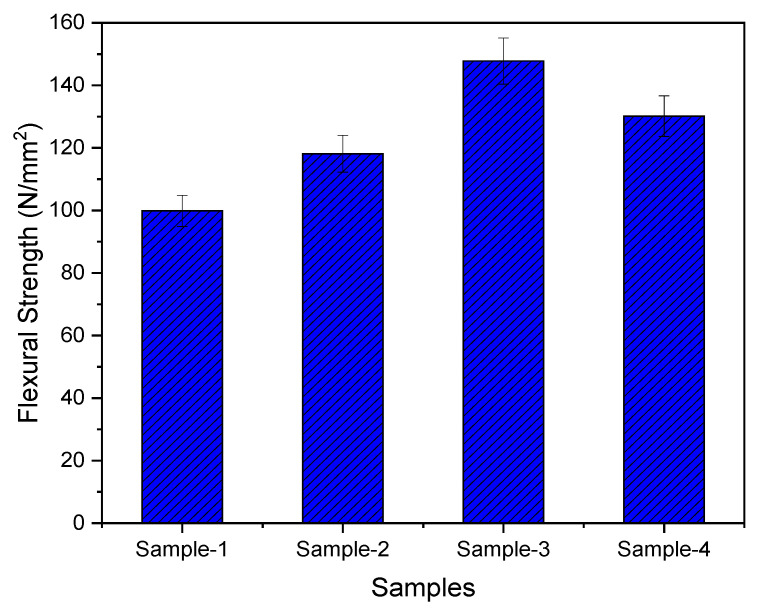
Flexural strength variation for non-functionalized (Sample-1: 0.1 wt.%) and functionalized (Sample-2: 0.1 wt.%; Sample-3: 0.5 wt.%; and Sample-4: 1.0 wt.%) MWCNT nanocomposites.

Figure 10 highlights the impact of the wt.% on the flexural strength of the MWCNT nanocomposites. The maximum value of flexural stress is 147.79 MPa at 0.5% of f-MWCNT, which is similar in comparison with the tensile stress at the maximum load. The effect of MWCNTs functionalization and variation in mixing ratios on the flexural strength is 15.78% (0.1 wt.%), 44.89% (0.5 wt.%), and 27.59% (1.0 wt.%) COOH-MWCNT in PMMA of the prepared nanocomposites.

The results of the present work compared with the published results are shown in Table 5 and Table 6. Regardless of the wt.%-MWCNT, the tensile and flexural strength values are clearly superior to the those reported in the studies in the literature. Small exceptions to this rule are observed in the case of tensile strength for a 0.1% wt.%-MWCNT, where the literature reports values up to 1.39% higher.

## 5. Conclusions

In the present study, polymer composites reinforced with different concentrations of carbon nanotubes (CNTs) were developed and experimentally investigated. The main mechanical properties of carboxyl functionalized multi-walled carbon nanotube (COOH-MWCNT) composites are significantly improved with the addition of f-MWCNTs. The tensile and flexural strength of MWCNT/PMMA nanocomposites increase with an increase in weight percentages of f-MWCNT up to 0.5 wt.%, and then it starts to decrease. Moreover, the adequacy of the process was validated, followed by an SEM examination in which uniform distribution was observed. Based on the results, we can conclude that the small amount of f-MWCNT is sufficient to upgrade the mechanical properties of nanocomposites. The property enhancement is due to the beneficial f-MWCNT properties, such as large contact surface area and phenomenal load absorbance. The fluctuation of properties at higher MWCNT percentages makes the nanocomposite harder, leading to both reduced flexibility and decreased tensile and flexural properties. Additionally, the novel mixing process, followed by a compression molding process, yielded a promising improvement of 25.41%, 35.85%, and 31.75% in tensile properties and 18.27%, 48%, and 33.33% in flexural properties for 0.1, 0.5, and 1.0 wt.% COOH-MWCNTs in PMMA compared to non-functionalized MWCNTs. The proposed nanocomposite is suitable for bone tissue engineering and for use as a scaffold material because the MWCNT phase meets the requirements of high strength, while PMMA provides the property of biodegradability.

## Figures and Tables

**Table 1:** Physical and mechanical properties of PMMA.

Properties	Description
Color	White
Particle size	48 micron
Tensile strength	45 MPa
Young Modulus	2855 MPa
Density	1.18 g/cm^3^

**Table 2:** Physical and mechanical properties of functionalized MWCNT.

Properties	Description
Color	Black
Length	0.5–5 μm
Diameter	10–20 nm
Purity	>98 wt.%
Density	2.60 g/cm^3^
Aspect Ratio (L/D)	183

**Table 3:** Mixing process parameters.

Trial	Factor Changed	Direction (Forward-F; Reverse-R)	Mixing Ratio (%wt.)	Speed (RPM)	Time (Minutes)	Visual Mixing Quality
1	Time	F & R	0.1	25	10	0
2		F & R	0.1	25	20	0
3		F & R	0.1	25	30	0
1	Speed	F	0.1	25	30	1
2		F	0.1	35	30	2
3		F	0.1	45	30	3
1	Time and Speed	R	0.1	35	10	1
2		R	0.1	35	20	1
3		R	0.1	45	30	3

**Table 4:** Compression molding process parameters.

Parameter	Description
Volume	270 cm^3^
Quantity of charge (mass)	102.6 cm^3^
Pressure of the molding process	700 kPa
Mold temperature: temperature range	260–285 °C
Cure time variables	6 h

**Table 5:** Comparison of tensile strength of MWCNT-reinforced PMMA nanocomposites.

CNT wt.%-MWCNT	Tensile Strength (MPa)	Tensile Strength (MPa)/Reference
0.1 non-functionalized	43.68	-
0.1	54.78	55.55 [49]
0.5	59.34	59.75 [50], 49.05 [51], 59.75 [52]
1.0	57.55	53.62 [52], 53.62 [15], 57 [51]

**Table 6:** Comparison of flexural strength of MWCNT-reinforced PMMA nanocomposites.

CNT wt.%-MWCNT	Flexural Strength (MPa)	Flexural Strength (MPa)/Reference
0.1 non-functionalized	98	-
0.1	118.1	110.93 [50]
0.5	147.79	112.60 [50], 121.61 [49]
1.0	130.15	111.62 [50]

## Data Availability

The data that support the findings of this study are available from the corresponding authors, [Chander Prakash and Emanoil Linul], upon reasonable request.
